# Supplementary material for: Using social media to quantify spatial and temporal dynamics of nature-based recreational activities
Source: PLoS One. 2018 Jul 16;13(7):e0200565. doi: 10.1371/journal.pone.0200565 (PMC6047793; doi:10.1371/journal.pone.0200565)
Supplement: S1 Appendix — Figure A. Flickr Active Users. Time series showing how many users posted at least one picture on Flickr (Flickr active users) in each month from January 2009 to April 2014. Figure B. Distance between multiple photographs by the same user. Distribution of the distance (in meters) between multiple photographs taken by each user on the same day. Figure C. Flickr visitor days. Spatial distribution of Flickr visitor days (FVD). Figure D. Variograms. Directional variograms of residuals of binomial GLM at the 5km resolution. Each panel shows semivariance in residuals in different directions: from North (panel 0) to North West (panel 320). Figure E. Time series wavelet power spectra. Wavelet power spectra of the two time series: a) CNP authority and b) Flickr time series. Colour code from dark blue (low values) to red (high values). White contour lines indicate significance. The shaded area near the edges in the graphs is the cone of influence, and indicates the range of the graph where the results are not reliable because of edge effects. Figure F. Survey visitor days. Spatial distribution of coastal survey visitor days. Figure G. Flickr visitor days normalised. Spatial distribution of coastal Flickr visitor days normalised by population size. Figure H. Bird watching density maps. Each panel represents the density of Flickr visitor days in a different year, from 2005 to 2015. The blue dots on the maps are the data. Different colours represent different density levels, from low (yellow) to high (red). Table A. QIC of the three alternative models. (DOCX) [file pone.0200565.s001.docx]

Figure A


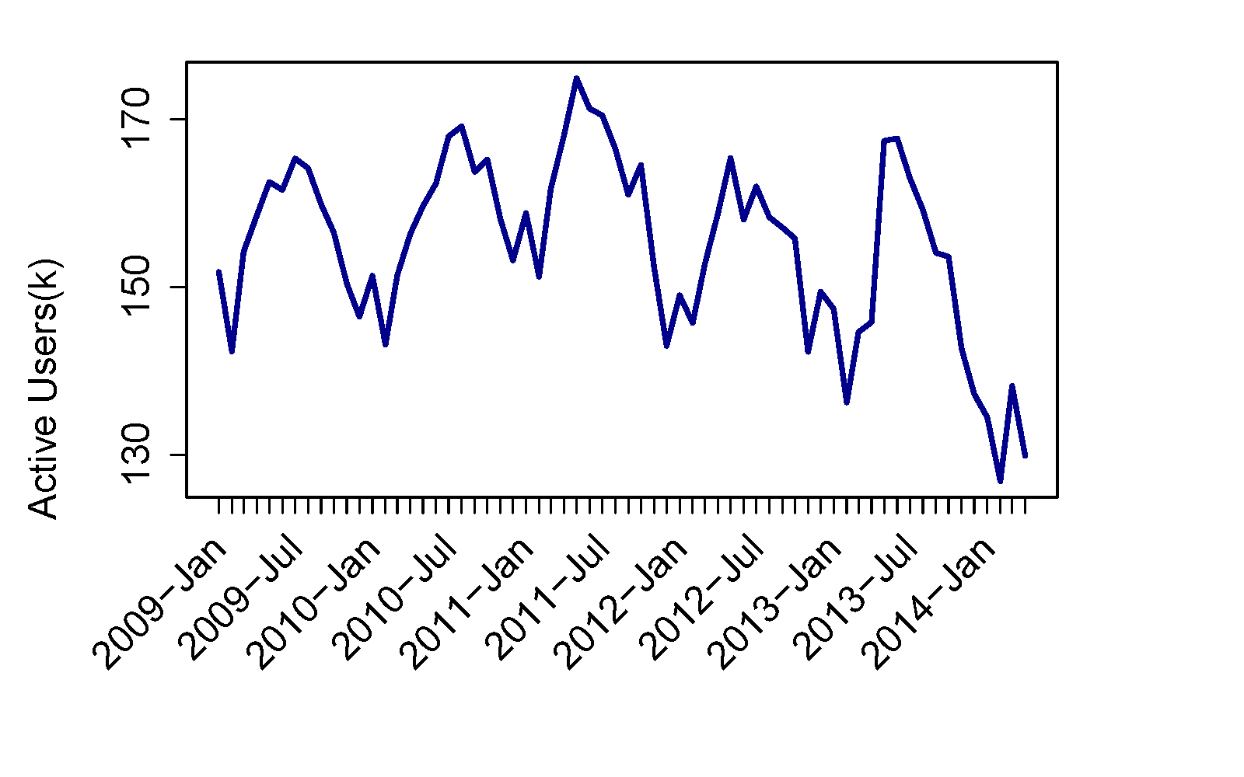


Figure B

**
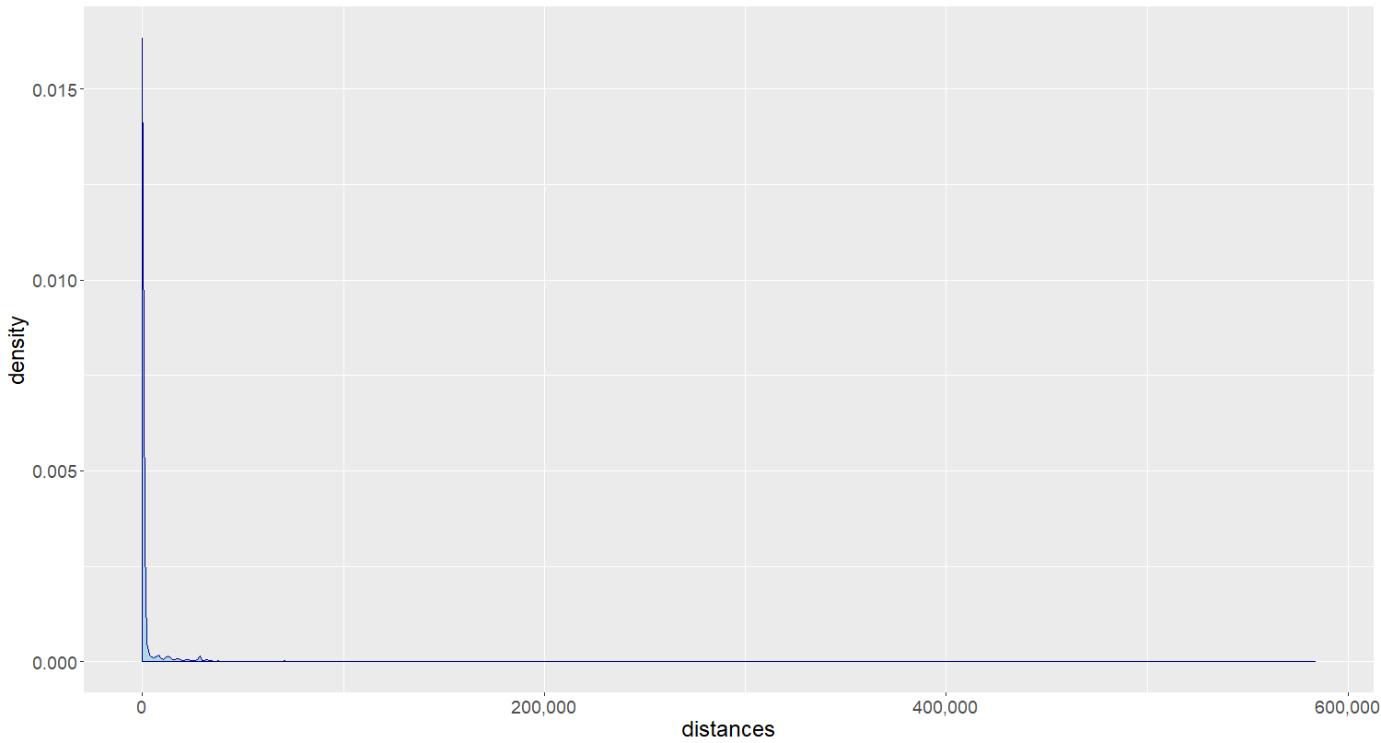
**

Figure C


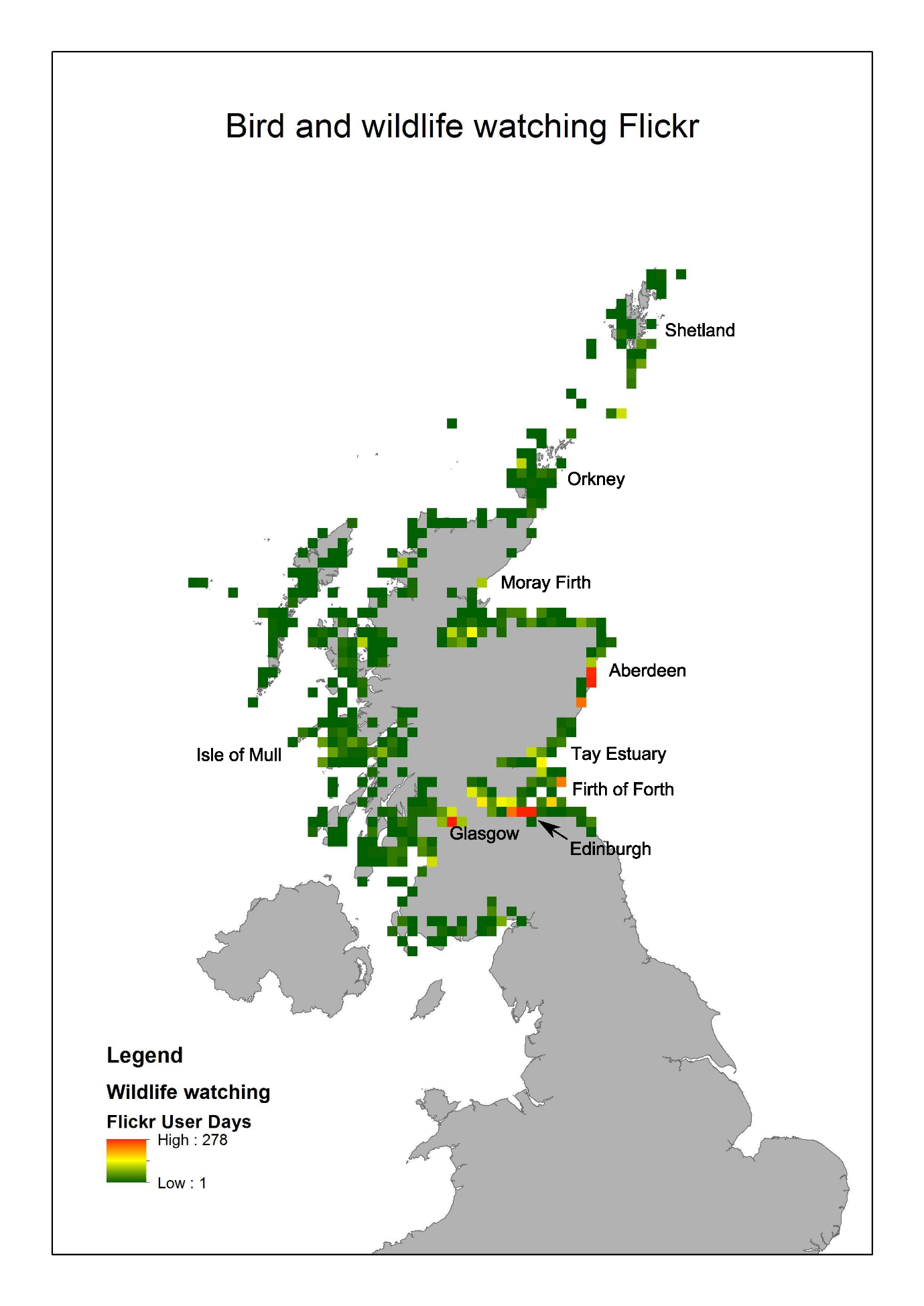


Figure D


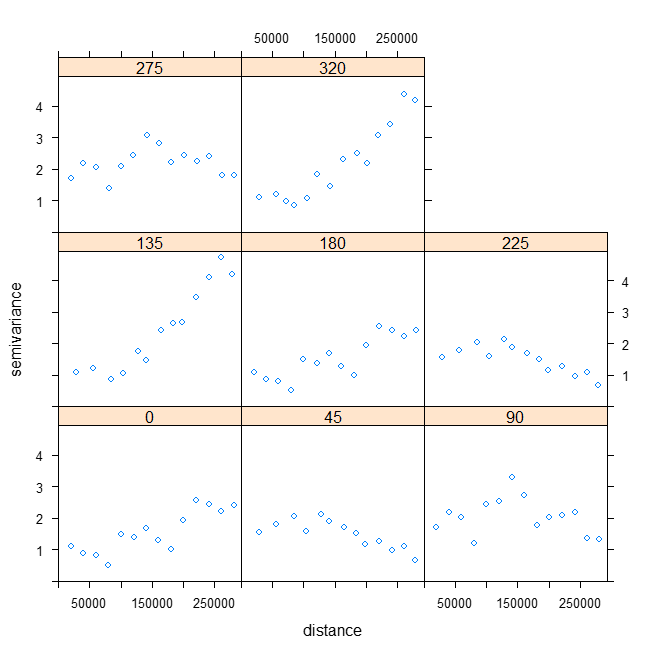


Figure E


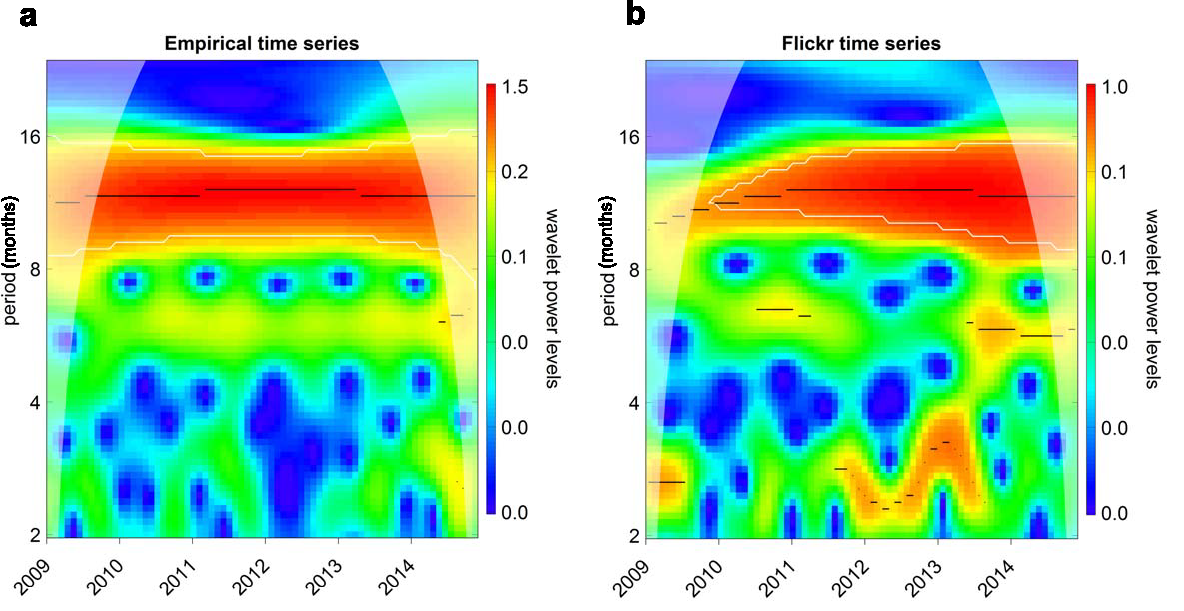


Figure F


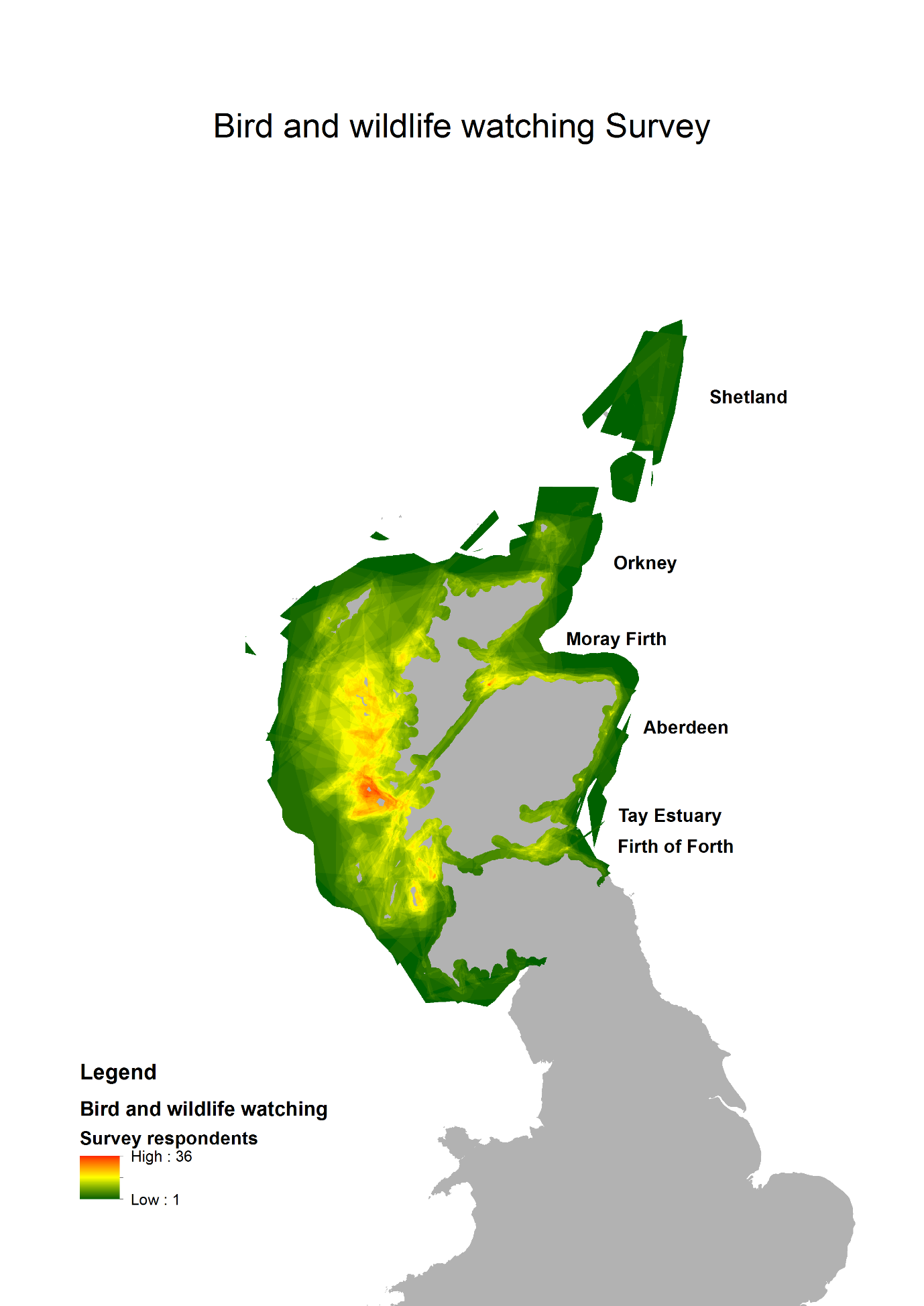


Figure G


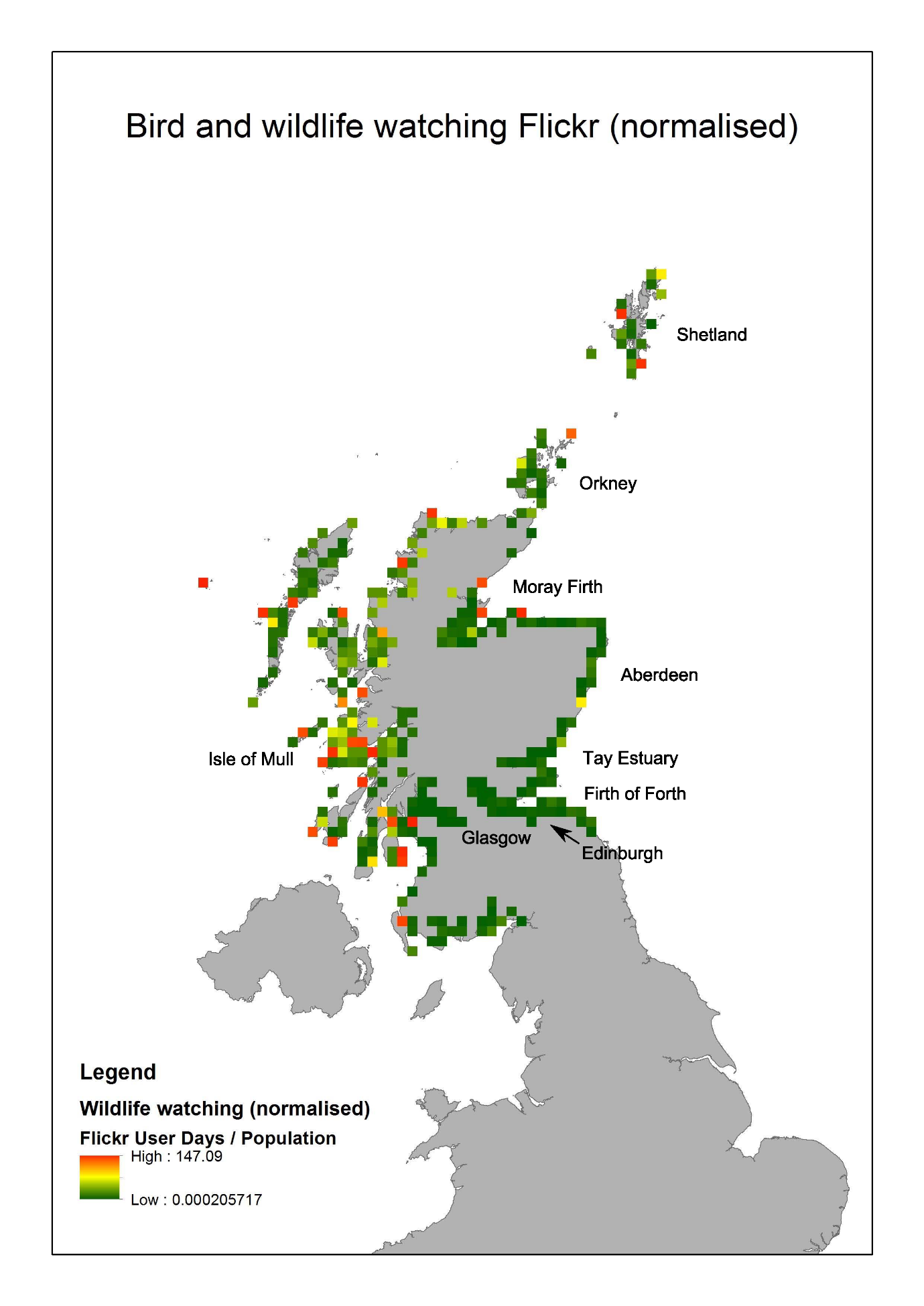


Figure H


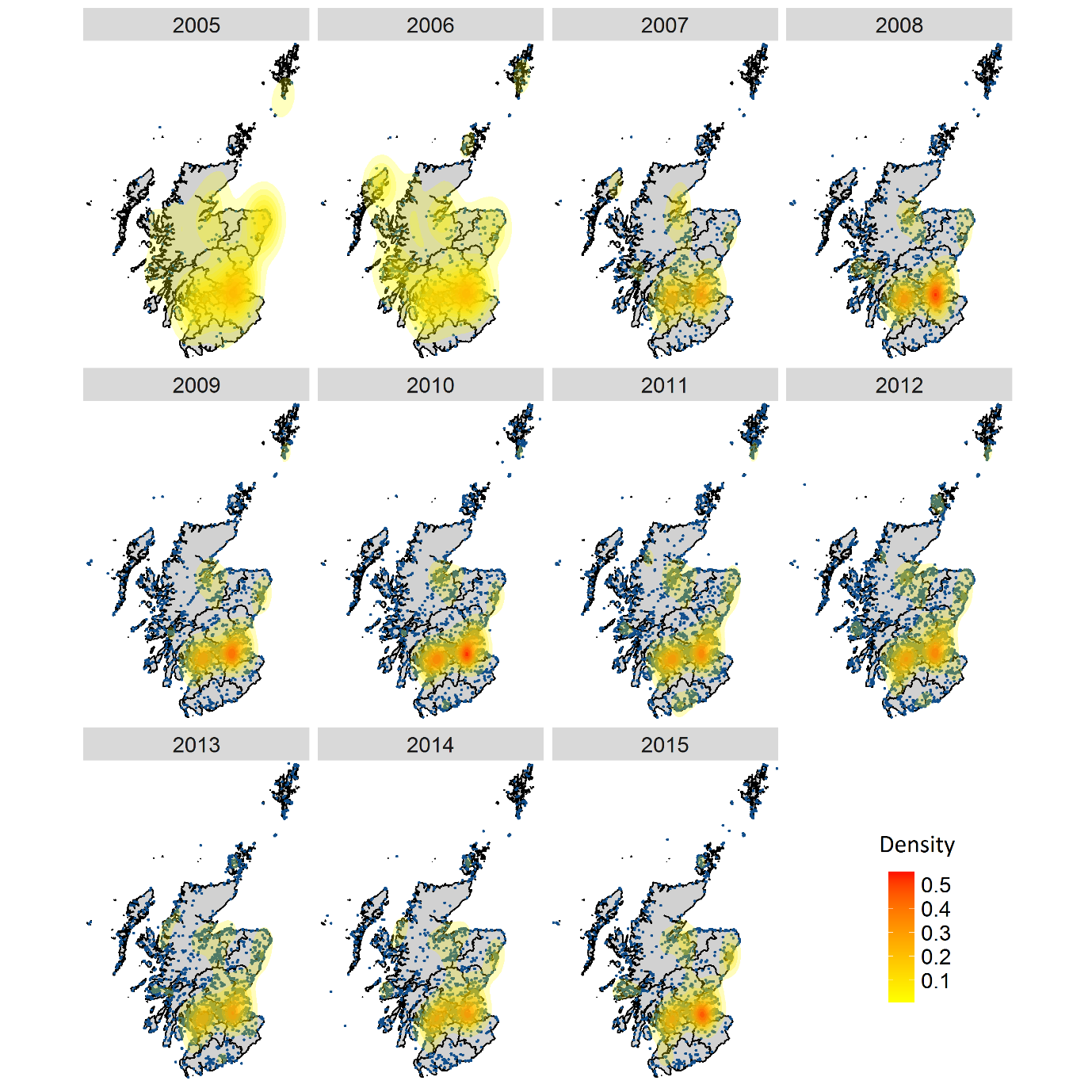


Table A

| Model | QIC | ΔQIC |
| --- | --- | --- |
| ~ CNP STEAM Visitor Days + Flickr Active Users | -25851.03 | 0 |
| ~ CNP STEAM Visitor Days | -25818.49 | - 32.54 |
| ~ Flickr Active Users | -25516.28 | -334.75 |
